# Extracellular Vesicles in the Fungi Kingdom

**DOI:** 10.3390/ijms22137221

**Published:** 2021-07-05

**Authors:** Marc Liebana-Jordan, Bruno Brotons, Juan Manuel Falcon-Perez, Esperanza Gonzalez

**Affiliations:** 1 Exosomes Laboratory, Center for Cooperative Research in Biosciences (CIC bioGUNE), Basque Research and Technology Alliance (BRTA), 48160 Derio, Spain; m.liebana98@gmail.com (M.L.-J.); brunaco2011@hotmail.es (B.B.); 2 Centro de Investigación Biomédica en Red de Enfermedades Hepáticas y Digestivas (CIBERehd), 28029 Madrid, Spain; 3 IKERBASQUE Basque Foundation for Science, 48009 Bilbao, Spain

**Keywords:** extracellular vesicles, microvesicles, exosomes, periplasmic vesicles, fungi, yeasts

## Abstract

Extracellular vesicles (EVs) are membranous, rounded vesicles released by prokaryotic and eukaryotic cells in their normal and pathophysiological states. These vesicles form a network of intercellular communication as they can transfer cell- and function-specific information (lipids, proteins and nucleic acids) to different cells and thus alter their function. Fungi are not an exception; they also release EVs to the extracellular space. The vesicles can also be retained in the periplasm as periplasmic vesicles (PVs) and the cell wall. Such fungal vesicles play various specific roles in the lives of these organisms. They are involved in creating wall architecture and maintaining its integrity, supporting cell isolation and defence against the environment. In the case of pathogenic strains, they might take part in the interactions with the host and affect the infection outcomes. The economic importance of fungi in manufacturing high-quality nutritional and pharmaceutical products and in remediation is considerable. The analysis of fungal EVs opens new horizons for diagnosing fungal infections and developing vaccines against mycoses and novel applications of nanotherapy and sensors in industrial processes.

## 1. General Concepts on Extracellular Vesicles

Extracellular vesicles (EVs) are membranous structures released by prokaryotic and eukaryotic cells during normal physiological functioning, whose secretion is altered in pathophysiological states. The biological importance of these vesicles relies on their capacity to mediate in intercellular communication by transferring information between cells [1]. 

The EVs have been widely characterized in mammalian cells and can be broadly divided into three main categories. The first type, exosomes, range from ~40 to 160 nm in diameter and are of endosomal origin. They are formed by the fusion of multivesicular bodies (MVBs) with the plasma membrane. After fusion, the intraluminal vesicles (ILVs) conforming MVBs are released from the cells, becoming exosomes. The second group, ectosomes, includes microvesicles, microparticles and large vesicles from ~50 nm to 1 µm in diameter. These vesicles pinch off the surface of the plasma membrane via outward budding. Finally, apoptotic bodies, ranging from 50 nm to 5 µm in diameter, are formed during plasma membrane blebbing in apoptosis. It has been widely accepted that these three EV subgroups have their specific features; however, there is no consensus on particular distinguishing markers [2]. 

Over the past few years, EVs have been isolated from nearly all cell types and biological fluids [3]. Their characteristic bilayer membrane, enriched in sphingomyelin, cholesterol, phosphatidylserine (PS) and glycosphingolipids, contributes to their stability in different extracellular environments, making them excellent shuttles for various molecules [1]. The EVs can carry many different macromolecules such as proteins, nucleic acids (both RNA and DNA), carbohydrates and metabolites that can act as signals [1]. The vesicles transport such macromolecules across biological barriers to the neighbouring and remote cells in specific organs or tissues via circulatory systems (blood, lymph). Thus, they can affect the function of recipient cells in multicellular organisms. The size, membrane composition and content of EVs are highly heterogeneous. They are dynamic and strongly dependent on the source cell, cell topography, state and environmental conditions. All these factors determine their functionality and target specificity. The same cell type may secrete different types of vesicles, depending on these factors [4,5].

Despite all the research already conducted in the field, the physiological purpose of generating these EVs remains largely unknown and needs further investigation. Most of the available information on their biosynthesis, composition and function comes from mammalian systems, presenting the EVs as novel players in cell communication and signalling as mentioned above. However, EVs released by other multicellular and unicellular organisms and cross-kingdom EV-based communication have also been described. For example, human–fungus and plant–fungus interactions have been reported. It has been suggested that fungal virulence is enhanced by EVs containing small RNA (sRNA). In turn, the plants can secrete EVs carrying sRNA to silence the fungal infection [6]. 

This review describes and discusses the properties of fungal EVs. Their functionality and the still unanswered questions are presented, including the cellular origin and biogenesis of EVs, transit mechanisms through the fungal cell wall and the importance and packaging of the cargo.

## 2. History of Fungal EVs 

Fungi form a diverse kingdom of eukaryotic organisms ranging from yeasts and moulds to mushrooms. Some species fill biological niches of basic decomposers, play a key role in recycling organic matter and others, such as many unicellular yeasts and filamentous fungi, are human, animal or plant pathogens. They can have a considerable impact on human lives. Widening our understanding of fungal biology is necessary to improve the treatment and prevention of fungal diseases. The emerging antifungal drug resistance may bring considerable epidemiological risks [7]. Only an effective control of pathogenic fungi that infect livestock and agricultural holdings can avoid substantial economic losses. However, fungi are also exploited in biotechnological processes and the food industry; thus, understanding their biology will help optimise the processes and prevent problems in end-product manufacturing. 

Unicellular fungi such as yeasts are key models in science since, as simple eukaryotes, they can reflect the basic cellular physiology and molecular biology of more complex eukaryotic organisms. In similarity with the bacterial and plant cells [6], fungal cells are protected by a thick, well-built cell wall composed of glycoproteins and carbohydrate polymers, with the kingdom-defining polysaccharide chitin [8]. This barrier protects them from the external environment and limits the exchange and communication in both directions. In this scenario, intercellular communication must be conducted, at least in part, via EVs (like in mammals).

**Figure 1:**
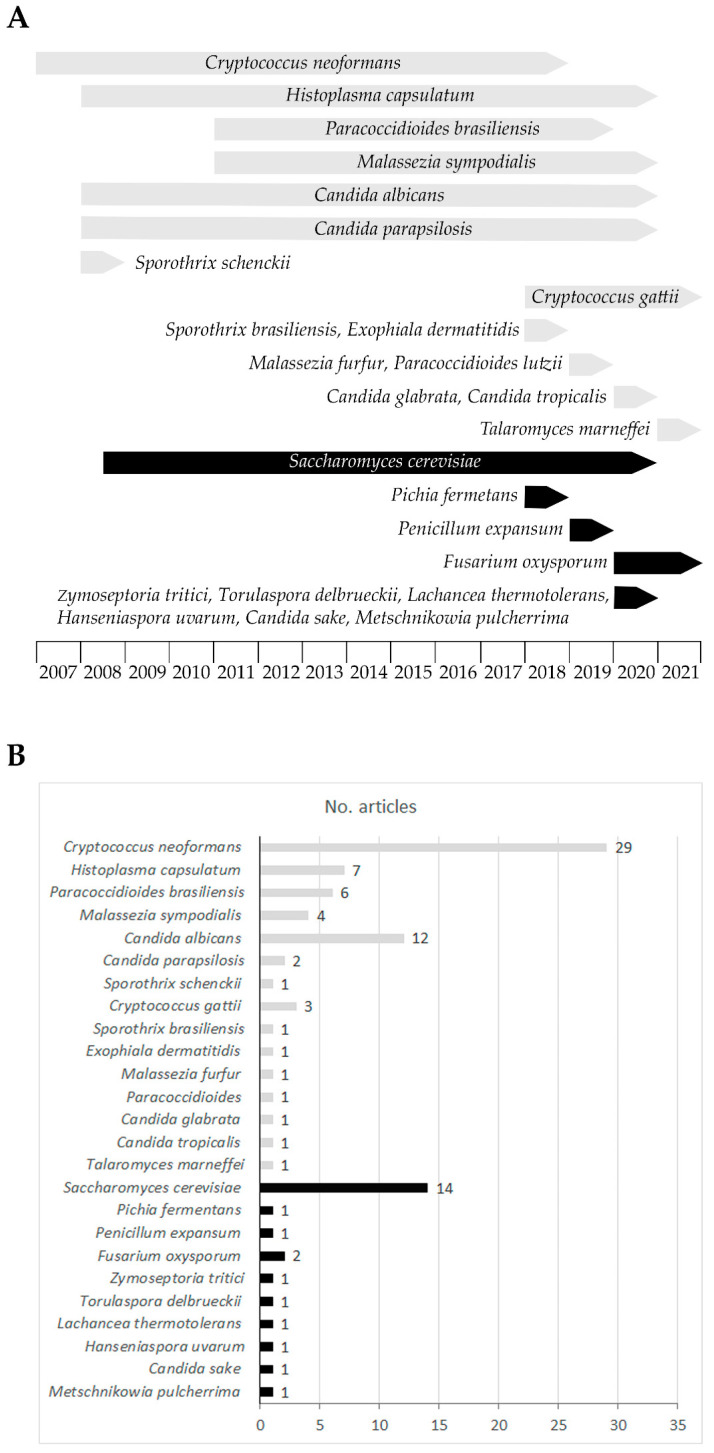
Summary of the research into fungal EVs. Research activity for each species is displayed (**A**) over time or (**B**) according to the number of articles published. In both panels, human pathogen species are represented in grey and those with industrial impact in black.

Research on fungal EVs is still in its infancy. They were first observed in the early 1970s, using the freeze-etching technique [9]. The interest in these vesicles later declined and reawakened at the beginning of the 21st century. A decade ago, fungal EVs were finally described and characterised in the study of macromolecules crossing the cell wall in the pathogen *Cryptococcus neoformans* [10]. This yeast secretes EVs containing glucuronoxylomannan (GXM), a component of the cryptococcal capsule and key lipids such as glucosylceramide and sterols. Since then, the EVs have been characterised in several other fungal species, both pathogenic and non-pathogenic (Figure 1) [11,12,13,14,15].

In addition, *C. neoformans*, EVs from other pathogenic fungi have been studied. Some of these fungi are species with particular prevalence in tropical and subtropical regions, such as *Histoplasma capsulatum* [16] and *Paracoccidioides brasiliensis* [17]. Others were the species with worldwide prevalence, *Malassezia sympodialis* [18] and the opportunist *Candida albicans* [19]. Recently, the opportunist *Exophiala dermatitidis* [20] and *Cryptococcus gattii* [21] have also attracted attention in the EV field, as well as *Sporothrix brasiliensis* [22], responsible for the emergent zoonosis in Brasil. EV secretion has also been investigated in fungus species with industrial interest. One example is *C. albicans* due to its role in the heavy metal accumulation and its possible application in remediation. *Saccharomyces cerevisiae* [11,23] and *Pichia fermentans* (used in the production of bread, beer and wine) and other less known fungi such as *Torulaspora delbrueckii*, *Candida sake*, *Hanseniaspora uvarum*, *Metschnikowia pulcherrima,* and *Lachancea thermotolerans* have been explored [24]. Other fungi with economic impact (infecting crops) like *Fusarium oxysporum* (affecting banana trees) have also been examined (Figure 1).

## 3. Characteristics and Composition of Fungal EVs 

In terms of molecular content, fungal EVs show strong similarities to mammalian EVs [25]. In both cases, the EVs transport several RNA species belonging to a wide range of functional categories, including messenger RNA (mRNA). They transfer several types of non-coding RNAs, such as transfer RNA (tRNA), ribosomal RNA (rRNA) and small non-coding RNAs like micro-RNA (miRNA), small nuclear RNA (snRNA) and small nucleolar RNA (snoRNA), among others (Table 1). The EVs are efficient vehicles for cell–to–cell transport of RNA since their structure protects the RNA from RNase degradation. Thus, this type of intercellular communication might affect the regulation of gene expression and alter important processes such as infection persistence or suppression of virulence factors [21,26].

Proteomic characterisation of EVs has detected proteins representing almost all functional categories, from pathogenesis and immune response to nutrition, metabolism, signalling and trafficking. These proteins come from different cellular compartments, such as the cell wall, plasma membrane, cytoplasm, mitochondria, vacuoles and even the nucleus (Table 1). Some of them are the proteins common in several fungus species or have orthologs in other species [15]. There are also some species-specific proteins, e.g., several septins found in *P. brasiliensis* EVs [14].

Lipid profiles of EVs from several fungus species, *C. neoformans*, *H. capsulatum*, *P. brasiliensis*, *C. albicans* and *C. gattii,* have also been characterised using different techniques such as thin-layer chromatography, electrospray ionisation or gas-chromatography-mass spectrometry. The analysis has shown some peculiarities in the membrane of these EVs (Table 2). The main sterol-derivatives detected are ergosterol [10,17,19,27] and lanosterol [17,19,27], whereas the most abundant neutral glycosphingolipid (GSL) in fungi is glucosylceramide (GlcCer) [10,19,27]. Moreover, several types of monohexosylceramide have also been found in different strains of *P. brasiliensis* [28]. Finally, there is an abundance of phospholipids such as phosphatidylethanolamine (PE), phosphatidylserine (PS), phosphatidylcholine (PC), phosphatidic acid (PA), phosphatidylinositol (PI) and phosphatidylglycerol (PG) [16,27,29]. The main fatty acids encountered are linoleic, oleic, palmitic, stearic and pentadecanoic acid [27,29].

Thus, the fungal EVs carry many common but also some kingdom-specific components. It should be noted that the composition of EVs from fungi depends on the species, cellular and environmental conditions and the experimental scenario. Such a composition makes EVs the active machines they are.

## 4. Biogenesis of Fungal EVs

### 4.1. Etiology of Fungal EVs

Both the EVs and periplasmic vesicles (PVs) have been described in fungi; however, it is still unclear whether the vesicles observed inside and outside the cell walls are the same. In *S. cerevisiae*, both types have similar rounded shape and size and similar protein composition [23,37]. PVs comprise two populations whose biogenesis is orchestrated by the nutrient environment. In particular, the subpopulation comprising the smallest vesicles along with their gluconeogenic enzymes content increase in response to glucose starvation [23]. However, the vesicles found in the extracellular space (EVs) have an ovoid or rounded shape in all species studied so far. They are mostly from 30 to 300 nm in size, although smaller and larger EVs are also detected. Like in PVs, their secretion is also affected by nutrient accessibility and growth conditions [31]. These findings support the idea that the nutritional environment can determine the profile of EVs secreted by a certain group of cells, reflecting an adaptation response to the medium. Indeed, the fungi can secrete EVs when cultured not only in liquid but also in solid media [32] and while forming biofilms [39,40]. In *C. albicans*, the EVs produced inside the biofilm are different from those formed by the free-living cells, both in size and composition [39] (Table 3). 

### 4.2. Crossing External Barriers 

**Figure 2:**
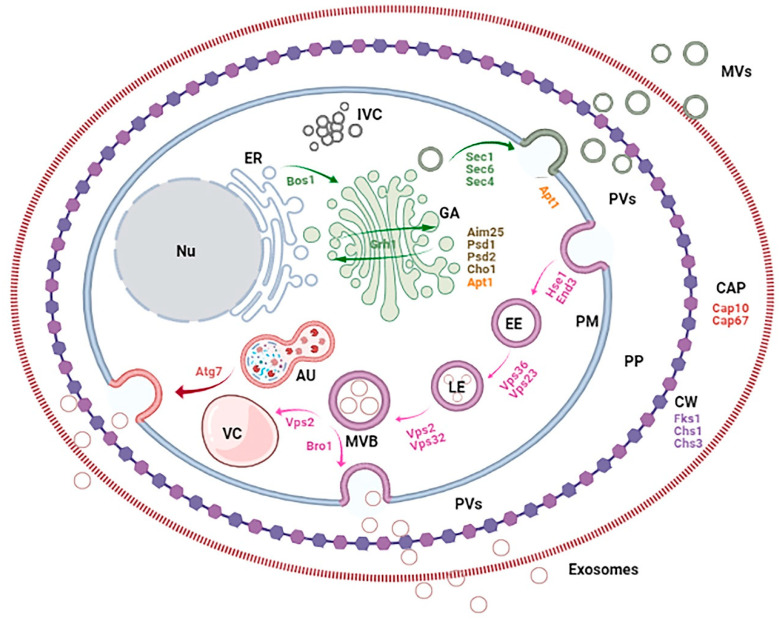
Fungal cell compartments, pathways and genes involved in EV biogenesis. The figure shows the conventional secretory axis (ER: endoplasmic reticulum; GA: Golgi apparatus; IVC: Intracellular vesicle clusters) and endocytic secretory pathway (EE: early endosomes; LE: late endosomes; MVB: multivesicular bodies; VC: vacuole), autophagosome (AU), cell barriers (PM: plasma membrane; PP: periplasm; CW: cell wall; CAP: capsule), vesicles (PVs: periplasmic vesicles and EVs, including MVs: microvesicles and exosomes) and nucleus (Nu).

The cell wall acts as a barrier in the final steps of EV release (Figure 2). *C. neoformans* and *S. cerevisiae* mutants with defects in cell wall remodelling and dynamics (such as *chs3* and *fks1* [49]) as well as *chs1* mutant in *S. cerevisiae* [44] show increased EV release, reflecting their weakened cell-wall phenotype. Caspofungin, an inhibitor of Fks1, can mimic this phenotype [44]. Turgor pressure could force the vesicles through the cell wall, given the viscoelastic properties of this barrier observed in *C. albicans* and *C. neoformans* [50]. 

In capsule-forming species like *C. neoformans*, the capsule will also work as a barrier in the EV release. An increase in EV release and virulence factors has been observed in the acapsular mutant *Δcap67* [10,32]. The elevated laccase and acid phosphatase virulence factors in the cell surfaces of *Δcap10* mutants might also be associated with increased EV secretion [51].

### 4.3. Trafficking EVs 

Proteomic and *in silico* studies of many fungal species had provided evidence on trafficking pathways involved in EVs biogenesis even before any experimental proof of their existence was obtained. Prediction of signal peptide cleavage sites in *S. cerevisiae* shows that some EV proteins target the ER and the conventional secretory pathway (involving the Golgi apparatus and the plasma membrane). Moreover, the GPI-anchored proteins are 4-fold enriched in the EVs compared to the genome background. This difference in their abundance suggests that GPI-anchor modifications tag the proteins to be transported in the vesicles [11]. Finally, *S. cerevisiae* EVs are also enriched in proteins containing AAA domains, which are implicated in membrane fusion, indicating a role of the membrane system in their biogenesis [44]. In the case of *P. brasiliensis*, 61% of the EV proteins have been rated as secretory, although only 10% have a predicted signal peptide. This result supports the hypothesis proposing various ways for proteins to reach or incorporate into the EVs. Regarding the putative GPI-anchored proteins, only 0.98% are represented in the EVs from *P. brasiliensis* [14]. In *C. albicans*, predicted signal peptides are detected in similar proportions, in 60% of proteins, of which, almost 20% are putative GPI-anchored proteins. The remaining 40% of proteins are not predicted to be secreted [38].

Poor understanding of the mechanisms of EV biogenesis is one of the main factors impeding the study of these vesicles, their associated pathways and regulators. Apart from *in silico* strategies, genetic studies have brought the most informative results. After many years invested in studying fungal EVs, the genetic and molecular evidence has shown, like in mammalians [52], the involvement of both conventional secretory pathway (endoplasmic reticulum (ER)–Golgi Apparatus (GA)–exocyst–plasma membrane axis) and the ESCRT-mediated MVB pathway (early-sorting-endosomes (ESEs)–late-sorting endosomes (LSEs)–MVBs pathway) in fungal EV biogenesis and cargo loading. There are also some additional "alternative" routes and regulators [53] (Figure 2). However, the relative contribution and biological significance of these pathways remain unclear. Mutations in the regulators of both conventional secretory and MVB pathways affect fungal EV composition and release kinetics (Table 4). Nevertheless, none of these mutants shows a complete abrogation of EV formation, suggesting that other pathways are implicated in vesicle generation. 

#### 4.3.1. Conventional Pathway 

In the conventional secretory pathway, transport protein Sec1, essential for the stimulation of membrane fusion, concentrates at the secretion sites. Sec4, a GTP-binding protein, regulates polarised delivery of vesicles to the exocyst at the plasma membrane. Transport protein Bos1, localised at the ER membrane, is necessary for the correct flow of vesicular transport from the ER to the Golgi. Mutations in the corresponding genes of *S. cerevisiae* affect the release of EVs [11] (Figure 2). In *sec4* mutants, EV secretion is decreased and delayed. The population size distribution changes: *sec4* mutant secretes vesicles that are distributed in two populations, from 80 to 120 and 400 to 550 nm (normally, the sizes range from 100 to 200 nm). Mutations in *SEC1*, *SEC4* and *BOS1* also alter protein abundance in the EVs without changing their composition. Mutations in *SEC1* and *SEC4* cause a decrease in the EV sterol content, accompanied by an intracellular accumulation of sterols [11] (Table 4). 

The exocyst, an octameric protein complex, is involved in the final step of the conventional secretory pathway, necessary for the polarised fusion of the exocytic vesicles with the plasma membrane (Figure 2). In *C. neoformans*, a knockdown of the SEC6 component suppresses the EVs release and results in an accumulation of large cytoplasmic vesicles and 100 nm vesicles in the cellular bud necks. This is accompanied by a reduction in laccase and urease activity and in the levels of glucan cell-wall polysaccharide. In contrast, other virulence factors such as extracellular phospholipase activity and capsule production are unaffected [54] (Table 4). This duality of SEC6 knockdown on virulence factors secretion suggests the existence of different secretory pathways that, in turns, can be associated with EVs or not.

#### 4.3.2. GRASPs, Autophagy, IVCs and Endocytosis 

The Golgi reassembly and stacking proteins (GRASP) tether the vesicles destined to fuse with the Golgi apparatus and sustain the Golgi structure to maintain the flow of proteins among the different cisternae [58,59] (Figure 2). Deleting the single ortholog of *C. neoformans* affects the EV release and sizes higher than 300 nm are no longer observed. Moreover, some of the EV mRNAs are enriched [35,55]. When *GRASP* deletion, GXM polysaccharide content is reduced and, consequently, the capsule formation [35,55]. As already mentioned for the *sec1* and *sec4* mutants [11], the EVs in the yeast lacking *GRASP* show reduced sterol levels; this is accompanied by intracellular sterol accumulation (Table 4). Autophagosomes have related to GRASP unconventional secretory pathway [60]; autophagy regulator Atg7 affects both physiological and pathogenic mechanisms in *C. neoformans* [61]. In agreement with this, *atg7* mutant is also affected in EV release and content [35]. 

The intracellular vesicle clusters (IVCs) are apparently not associated with the conventional secretory pathway [62]. However, as some of the IVCs resident proteins in *S. cerevisiae*, such as gluconeogenic enzymes FBPase, Gapdh and Vid24p, are associated with EVs, the role of this compartment in PVs biogenesis has been examined. Nevertheless, reducing the IVCs activity does not affect the number of EVs in the periplasmic space [62]. 

The response of PVs to glucose starvation is regulated by endocytosis, involving the actin cytoskeleton-regulatory complex **protein End3.** This is accomplished through the sequential recruitment at endocytic sites of the **proteins** driving the cargo sorting, membrane invagination and vesicle release [23]. This mechanism has not been explored in the EVs; its further examination might shed light on the similarities between the periplasmic and extracellular vesicles (Table 4).

#### 4.3.3. The Non-Conventional Secretory Pathway 

In the non-conventional pathway involving the MVBs (Figure 2), Vps27 protein (a member of the ESCRT-0 complex) is required for sorting ubiquitinated membrane proteins into intraluminal vesicles before vacuolar degradation. Vps23 is a core component of the ESCRT-I complex, associated with the ubiquitin-dependent sorting of proteins into the endosome. Vps36, a member of the ESCRT-II complex, is involved in interactions with the ESCRT-I. Snf7/Vps32 and Vps2 are the essential members of the ESCRT-III and take part in the sorting of transmembrane proteins into the MVB pathway. Finally, Bro1 is a cytoplasmic class E VPS factor coordinating deubiquitination, recruited to MVBs by interacting with the ESCRT-III subunit Snf7/Vps32 [63]. 

In *C. neoformans vps27*(0), an accumulation of MVBs and vacuole fragmentation have been observed, while the cytoskeleton is not affected. The EVs in this mutant are larger (diameter over 200 nm) but their lipid composition is unaltered [49]. In *S. cerevisiae*, *vps23* (I) and *vps36* (II) knockouts result in a significant increase in the proportion of large EVs (150–500 nm) and a reduction in the relative abundance of vesicles between 30 and 150 nm in diameter [44]. Mutant strains *vps23* (I)*, vps 36* (II), *snf7/vps32* (III) and *vps2* (III) produce EVs with lower protein content than the vesicles from wild type cells and *vps27* and *bro1* mutants [11,44]. Similarly, the *vps23* (I)*, vps 36* (II) and *vps2* (III) mutations alter the EV protein composition, while the *vps27* (0) and *bro1* mutants do not affect it [44]. Notably, the EVs from mutants of ESCRT components are enriched in proteins containing AAA domains, which are implicated in membrane fusion [11] (Table 4). 

Interestingly, the EVs from *S. cerevisiae vps23* (I) and *vps36* (II) mutants are gained in cell-wall related proteins such as chitin synthase Chs3. Increased levels of the subunit of the major 1,3-beta-glucan synthase Fks1 have been found not only in the EVs from *vps23* (I) and *vps36* (II) but also from *vps 2* (III) mutants [44]. In *C. neoformans* *vps27* mutant, the capsule is reduced and virulence factors, like laccase, are accumulated mostly in EVs rather than in the cell walls. Surprisingly, the *LAC1* gene that codify for laccase is downregulated to compensate for trafficking defects. In this *C. neoformans vps27*, a reduction in urease activity and alterations in melanin production is also produced [49]. In *C. neoformans*, the absence of cyclin Cln1 results in an increase in GXM deposition and an apparent increase in the EV release [57]. Intriguingly, the absence of laccase activity in the cells is accompanied by *LAC1* downregulation [64], such as in *C. neoformans vps27,* suggesting a feasible trafficking of this enzyme to EVs and a connection between cyclin Cln1 and the non-conventional secretory pathway. 

When the yeasts are cultured as biofilms, the mutations in ESCRT subunits cause alterations in EV secretion reducing the matrix polysaccharide production (glucan and mannan) [39] (Table 4).

### 4.4. Lipid Homeostasis

Lipid asymmetry is important for cellular homeostasis, including regulation of membrane curvature and vesicle trafficking (Figure 2). Enzymes such as flippases, floppases and scramblases are essential for the translocation of phospholipids and their proper disposition [65,66]. Aminophospholipid translocases (APTs or APLTs) flip specifically phosphatidylserine (PS) and phosphatidylethanolamine (PE) from the external face to the inner side of the membrane [67]. In *S. cerevisiae*, five APTs have been described and characterised as components of the Golgi apparatus (Drs2p, Dnf1p, Dnf2p, Dnf3p and Neo1p); they are all involved in vesicular traffic [68]. Indeed, in *C. neoformans, apt1* mutants show Golgi structures concentrated in the centre of the cell instead of the typical peripheral distribution, aberrant vacuole morphology and formation of gigantic multivesicular body (MVB)-like compartments [69]. Interestingly, the size of EVs secreted in this mutant is also altered. *C. neoformans* secretes two populations of EVs, from 10 to 150 nm and from 400 to 1000 nm in diameter. In *apt1* mutants, the largest EVs are distributed in a narrower range of 400 to 600 nm. Moreover, defective intracellular GXM synthesis in mutant cells give rise to a significantly lower content of GXM polysaccharide in EVs, which results in an attenuated virulence and defective oxidative stress response in the host [61,69]. The absence of the scramblase Aim25 also results in disorganised membranes; the cells lack the typical vacuoles and show aberrant membranous structures [32]. In *C. gattii*, knockout mutation in *Aim25* decreases the production of EVs. The EVs from the *aim25* mutant are also larger than normal and their RNA content is altered. Physicochemical properties are also altered, resulting in a more efficient release of GXM from EVs to the capsule, thus increasing its dimensions. The data described above demonstrate the importance of phospholipid translocation in membrane organisation and EV secretion (Table 4). 

It must be noted that not only the translocation of the phospholipids but also their synthesis is essential for the biogenesis of the EVs. Phosphatidylserine synthase (Cho1) and phosphatidylserine decarboxylases (Psd1 and Psd2) are involved in phosphatidylserine and phosphatidylethanolamine biosynthesis, respectively [70]. In *C. albicans*, a double mutant *psd1/psd2* produces much larger EVs. In *cho1* and *psd1/psd2* mutants, the EV phosphatidylcholine and protein contents are distorted. Several EV-associated virulence factors are absent in this scenario, such as phospholipase Plb3 and adhesin Sim1 in *cho1* mutant and proteinase Prd1 in the double mutant *psd1/psd2* [29] (Table 4).

### 4.5. Other Players in EV Biogenesis

Other proteins also are implicated in EV biogenesis, involving mechanisms poorly understood. Decreased expression of a 14-3-3 protein, normally abundant in cryptococcal EVs, leads to reduced EV release [71]. Mutant phenotype also involves lighter and shorter capsule, lower EV GXM, protein content and activity of laccase and acid phosphatase [72]. Blocking of Hsp60 by monoclonal antibodies in *H. capsulatum* leads to fluctuations in EVs size, release and protein loading [43,73].

## 5. Functionality and Biological Implications of Fungal EVs 

**Figure 3:**
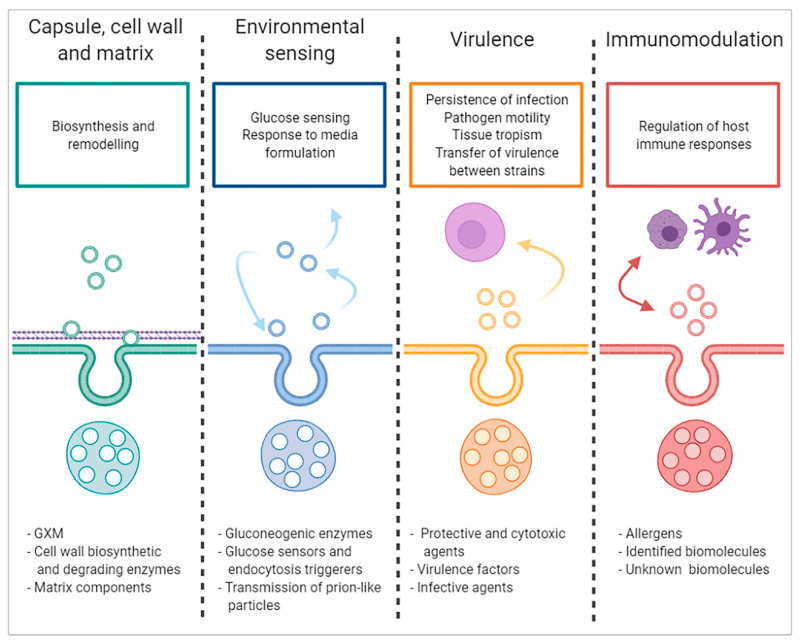
Functions of fungal EVs. Descriptions of the functions (Capsule, cell wall and matrix synthesis; Environmental sensing; Virulence and Immunomodulation) are shown at the top of the figure and the corresponding responsible EV components at the bottom.

The functions of fungal EVs comprise a wide spectrum of responses to internal and external stimuli. These responses must be understood not only in the context of individual cells but also in the process of coordinating fungal activity in a community (Figure 3).

### 5.1. Protective and Defensive Structures: Capsule, Cell Wall and Matrix

The first study reporting EVs in a fungal experimental model (*C. neoformans*) demonstrated the presence of glucuronoxylomannan (GXM), the major component of the cryptococcal capsule. The GXM has been detected in the lumen of the vesicles found in the extracellular space and those attached to the cell wall. GXM is transferred in these vesicles from the cytoplasm to the cell wall. The vesicles are released from the cells and then taken up by the producers themselves and neighbouring cells, aiding in the capsule assembly and maintenance [10,32]. In agreement with this, enhanced EV release could be responsible for the increased GXM deposition that results in enlarged capsule phenotype in the *C. neoformans cln1* mutant [57].

Cell wall-remodelling enzymes are a part of EV cargo in *S. cerevisiae (*Fks1 and Chs3) [44], *H. capsulatum* (Chi1) [16] and *Aspergillus fumigatus* (Fks1, Gel and Bgt) as well [74]. The EVs from *P. brasiliensis* also carry glycoconjugates containing α-galactosyl epitopes. These glycoconjugates are stored in intracellular vacuoles and might travel to the cell wall utilising the MVB pathway [17]. Thus, the available data indicate that EVs have a role in cell wall homeostasis. Indeed, mutating components of ESCRT machinery in *S. cerevisiae* reduces the release of EVs but increases their Fks1 and Chs3 levels and stimulates cell wall synthesis [44]. 

It has been proposed that the EVs might take part in matrix biogenesis, development and maintenance [39]. *C. albicans* growing in biofilm communities secretes EVs that carry proteins involved in extracellular matrix structure and biogenesis, specifically polysaccharide modification enzymes and polysaccharides (i.e., mannan and glucan). 

### 5.2. Pathogenesis

Cell wall and capsule structures protect the cell from adverse environmental conditions and host immune agents; they are considered the primary pathogenic elements for most fungal species as they ensure their survival [44]. Thus, the participation of fungal EVs in the biogenesis and maintenance of the capsule and the cell wall may be considered a pathogenic mechanism itself. In *S. cerevisiae*, EVs from mutants in ESCRT machinery rescue the cells from antifungal treatment because of their activity in cell wall synthesis [44]. In *C. neoformans* [10] and *C. gattii* [21,32], the EV GXM-related capsule synthesis constitutes a protective rather than a virulent mechanism, allowing the yeast to survive in the cells of its usual environmental predator, the amoeba *Acanthamoeba castellanii* [75]. When the release of EVs is blocked in *C. albicans* forming biofilm-protected communities, a strong sensitivity to several antifungal drugs is observed as the matrix synthesis fails [39]. 

First reviews of fungal EV studies have classified them as *virulence bags*. The concept has its origin in early proteomic studies of EVs from *C. neoformans* and *H. capsulatum* [76], in which virulence factors such as chaperones (e.g., Hsp60 precursors, Hsp70 and Hsp30), catalase B and superoxide dismutase have been identified. The enzymes laccase, urease, phosphatase and catalase were shown to be active [13]. Since then, other molecules involved in the pathogenicity of other fungi species have been described, such as melanin in *E. dermatitidis* [20], GlcCer and sterols [10,17,19,27]. GlcCer plays an important role in fungal cell division, alkaline tolerance, hyphal formation and spore germination, being it is a key regulator of pathogenicity [28]. In the *C. neoformans cln1* mutant, boosting in EV release is accompanied by an increase in their sterol content and strengthened virulence [57]. This strongly suggests an active role for the EVs in the virulence regulation (Table 1 and Table 2). 

On a more physiological level, *E. dermatitidis-*derived melanised EVs may mediate the neurotoxicity associated with this neurotropic black yeast [20]. In *Zymoseptoria tritici*, a wheat pathogen, the EVs are involved in switching from a yeast-like form of growth to a filamentous mode, responsible for pathogenicity in plants [77]. In *S. cerevisiae*, both the PVs and are involved in prion transmission. Interestingly, both native and aggregated states of the fungal prion Sup35p can be EV-packaged, maintaining their infectivity [37,47]. Moreover, EVs from fungi pathogens infecting plants, as *Fusarium oxysporum*, are able to induce a phytotoxic response [36].

### 5.3. Immunomodulation

There has been a lot of interest in the ability of EVs to regulate the host immune system; many cases of immunogenic molecules found in the EVs have been described (Table 1 and Table 2). Some EV-associated molecules (e.g., *S. brasiliensis* [22]) can induce immunological responses and can become allergens, as reported for *M. sympodialis* [18,42]. *P. brasiliensis* secretes the immunogen alpha-linked galactopyranosyl (α-Gal) associated with EVs [17]. Lipids such as GlcCer [10,19,27], which exhibit a characteristic and specific structure in comparison with the form found in mammals and plants and ergosterol [10,17,19,27] are also immunomodulators [28]. Hgt1p, a high-affinity glucose transporter from *C. albicans* EVs, can also act as a complement factor H (FH) binding molecule, mimicking human cells and, thus, evading the immune response and resulting in increased virulence [46].

Several types of immune cells can be modulated by the yeast EVs. Some early studies have shown that *C. neoformans*-derived EVs can alter the functionality of murine macrophages *in vitro*, achieving cross-kingdom communication and modulation of host cell response during infection [78]. In another example of such interactions, *P. brasiliensis* EVs can induce M1 polarisation of murine macrophages [79]. This triggers the production of proinflammatory mediators (e.g., NO, IL-12, IL-6, TNF-α) and an increase in the fungicidal activity, helping the host to limit the infection. In contrast, *H. capsulatum* EVs inhibit phagocytosis in macrophages as well as intracellular yeast killing by these innate immunity cells, thus promoting survival of the fungus and persisting infection. This effect has been observed during a low-level immune response simulated in vitro, suggesting a bidirectional communication between the host and pathogen cells [73]. 

The activity of other immune cells, such as monocytes, can also be modulated by fungal EVs [42]. Dendritic cells (DCs) stimulated with *S. brasiliensis* EVs increase phagocytosis and cytokine production. However, in contrast to EVs from *C. neoformans* [78] and *P. brasiliensis* [79], this results in a rise in the fungal load. *In vivo* assays have confirmed that, effectively, the EVs enhance fungal burden and skin lesions in murine models [22]. Other evidence of immune system modulation has been obtained for the skin-colonising commensal yeast *M. sympodialis*. The EVs from this fungus (designated MalaEx) can activate keratinocytes by augmenting the *ICAM-1* expression [48] and IL-4 and TNF-α responses in DCs [18]. 

The MalaEx might have a role in atopic eczema. The MalaEx EVs release digestive enzymes to break down sebaceous lipids on the host skin, producing irritant unsaturated free fatty acids. This activity can lead to various skin disorders associated with this lipid-dependent yeast genus [42]. Similarly, the EVs derived from *Trichophyton interdigitale* can induce proinflammatory cytokine release (e.g., NO) from macrophages and keratinocytes. These observations suggest that fungal EVs from sources other than the yeast cells can also have some pathogenic functions [80]. 

Interestingly, EVs from *C. gattii* play an active role in the division of labour of this pathogen. During infection by outbreak strains of *C. gattii*, the host reactive oxygen species induce tubular mitochondrial phenotype in some of the phagocyted fungal population. These altered population protect the normal cells, fostering their intracellular proliferation, leading to enhanced pathogenesis within the outbreak lineage [81]. EVs from virulent cells can diffuse over significant distances, become rapidly internalised by macrophages and then trigger increased rates of proliferation in cryptococci residing within the phagosome [21].

### 5.4. Host Modulation of Fungi Pathogenicity

Host can also modulate the functionality and pathogenicity of yeast EVs via the immune system. Exposing *H. capsulatum* to monoclonal antibodies significantly alters its EV characteristics, e.g., their size, protein loading and activity of some enzymes related to virulence, such as laccase, catalase and phosphatase. This can decrease the pathogen virulence and change the outcome of infection in mice [43]. However, pathogen modulation is not exclusive to mammals. Regente et al. have demonstrated that sunflower EVs contain defensive agents that promote morphological changes and cell death in the phytopathogenic fungus *Sclerotinia sclerotiorum* [82]. 

In this scenario, the EVs must be stable to be functionally successful. Ergosterol is a pivotal component of fungal EVs since serum albumin can bind to it, destabilising the EV membrane. In the course of infection, albumin can compromise the integrity of the fungi EVs, causing their rupture and the consequent release of potential immunomodulators [83].

### 5.5. Nutrition and Environmental Sensing

Proteomic studies of fungal periplasmic and extracellular vesicles have demonstrated they carry proteins related to nutrient exploitation, from glucose to phosphate to iron (Table 1), indicating that such vesicles might take part in environmental sensing. In *S. cerevisiae*, small PVs carrying the gluconeogenic enzymes FBPase, Pck1p, Mdh2 and Icl1p are secreted under conditions of glucose starvation and internalised in the presence of glucose [23,84]. Thus, the export of prion-like proteins in PVs is dependent on glucose availability [37]. Interestingly, the EVs from *C. albicans* carry Hgt1p, the high-affinity glucose transporter; this might be associated with glucose sensing in the extracellular environment and, in turn, with induction of Hgt1p synthesis and its transport to the plasma membrane [46].

Furthermore, the EVs change their composition depending on growth conditions, supporting that they might participate in nutrient sensing and adaptation to the medium. Some of the many examples of research in this field are the study of *C. albicans* in biofilm culture [39,40], *C. gattii* in solid media [32], *P. fermentans* in two liquid formulas [31] and more recently, an exhaustive study of *H. capsulatum* cultured under four different conditions [85].

## 6. EVs Isolation and Characterization Procedures

Environmental conditions affect the production of fungal PVs and EVs, which is species-, strain- and application-dependent. The variables to be considered are the temperature, timing, nutritional conditions, shaking of yeast cultures, liquid or solid media or biofilm format (summarised in Table 3).

For purification of EVs, most authors follow the gold standard established for mammals, based on differential centrifugation and concentration by ultracentrifugation. As the first step, the general protocol recommends low-speed centrifugation to remove the whole cells and small debris. This consists of first centrifugation at 4000× *g* for 15 min at 4 °C, followed by the collection of the supernatant and further centrifugation for 15–30 min at 15,000× *g* at 4 °C to remove or separate large vesicles and aggregates (Step 1). The supernatant is usually concentrated by ultrafiltration (20-fold concentration using a cut-off of 100 kDa) (Step 2). Sometimes, further serial centrifugations are performed, as in Step 1 and the supernatant filtered through filters with a variable pore size (from 0.2 to 0.8 µm) (Step 3). Finally, several serial ultracentrifugation steps (100,000× *g* for 1 h at 4 °C) are executed to precipitate and wash the EVs [Step 4]. All such centrifugation-based procedures found in the literature are modifications of the method used by Rodrigues et al., 2007, for the first-ever fungal EV purification. They are now thoroughly adapted to the specific needs.

If high-purity EVs are required, a density gradient (sucrose or iodixanol) [10,18] or size exclusion chromatography can be performed [86]. A noteworthy option for specific subpopulations management is the immunocapture method [10,18]. Generally, this is limited by the shortage of surface markers. However, a recent study has reported that Sur7 and Evp1, claudin-like proteins from the Sur7 family, might be useful as EV surface markers in fungi [40]. These methods have similar limitations for the fungal and mammalian EV sources; it is not possible to separate the heterogeneous EV populations from non-vesicular contaminants.

Characterisation of isolated EVs is usually conducted employing transmission electron microscopy (TEM), nanoparticle tracking analysis (NTA) and immunoblotting (WB). TEM is used to visualise the EVs, their spatial distribution and shape. The cryo-EM method gives excellent results since the cells and EVs retain their innate state and structure details. NTA quantifies the EVs in the sample and obtains their size distribution within the population, similarly to the dynamic light scattering technique (DLS). The WB allows protein detection by using specific antibodies. In addition, WB, other immunodetection methods can be used, such as immuno-gold labelling combined with electron microscopy and antibody-coupled bead flow cytometry analysis. These techniques can be combined with other methods mentioned above to widen the spectrum of variables that can be studied and obtain more reliable results [87,88].

## 7. Discussion

Fungi are of great interest in many different areas, from biological research to human, animal and plant health and various industrial fields. The yeasts have been widely used as the eukaryotic model because their cellular activities and mechanisms are common or closely related to the organisms placed higher on the evolutionary ladder. Recently, extracellular vesicles have emerged as a novel intercellular communication model. They have been discovered in mammals, but all kingdoms and species studied so far have shown similar cell–to–cell communication features. As discussed here, all fungi species, whether pathogenic (such as *C. neoformans*, *H. capsulatum*, *P. brasiliensis*, *M. sympodialis*, *C. albicans*, *S. brasiliensis*, *C. gattii*, *E. dermatitis* or *F. oxysporum*) or industrially relevant (e.g., *S. cerevisiae* and *P.fermentans)*, secrete vesicles of this type (Figure 1). As expected, the EVs in the fungi are involved in the delivery of a wide variety of molecules to the extracellular milieu, such as proteins, nucleic acids, lipids and polysaccharides related to EV functionality (Table 1 and Table 2). Thus, in a natural environment, intercellular communication via the EVs may help these organisms to survive under cell wall-damaging conditions (such as saline stress), conduct nutritional sensing or maintain their virulence (Figure 3). 

In the fungi, like in mammals, both conventional ER–GA–plasma membrane secretory axis and the endocytic secretory pathway are involved in the EV biogenesis (Table 4). In fact, some secretory components are highly conserved throughout the Eukaryota domain. In the yeast, the non-classical components such as Golgi reassembly and stacking proteins (GRASP) and autophagosome have also been associated with EV secretion. The mechanisms involved in the organisation and homeostasis of lipids have a key role. This highlights the underlying complexity of the regulation of EV secretion. In *S. cerevisiae*, some vesicles located in the periplasmic region have been described as PVs; however, they might have been the EVs on their way to the extracellular space (Figure 2). It is still unknown whether the EV secretion could be switched off and what would be the key factor in such a process. Clearly, there are many details of EV biogenesis, identification and secretion that must be investigated and understood.

## 8. Current Limitations

Although our understanding of fungal EV biology is advanced, we are still hampered by several limitations that need to be addressed. Some of them are common to all biological systems. For example, the physiological relevance of many studies might be questioned as the stoichiometry of EV release remains elusive. Experimental analysis normally does not reflect whether EVs are secreted constitutively under real-life conditions or not. Moreover, none of the currently available protocols can isolate pure EVs or even properly separate the subtypes. Distinguishing true vesicles from cell artefacts is often difficult and reliable EV markers are scarce. Further research is necessary to overcome these difficulties.

## 9. Future Perspectives

The EV research is strongly stimulated by the potential of these vesicles as diagnostic and therapeutic tools. As the EVs can be isolated from biological fluids, they make excellent targets for future liquid biopsy strategies. The components of fungal EVs could be used to diagnose both simple and complex infections or those associated with other conditions. The infection progression also could be monitored and used to make therapeutic decisions. Moreover, fungal EVs can modulate the immune system of the host and affect the disease progression. In this field, the utilisation of fungal EVs in biodelivery-based therapies and vaccine technologies looks very promising. Some bacterial EVs have been already evaluated as adjuvants in a meningococcal vaccine [89]. In the clinical field, fungal EVs could also be used to produce various vaccines (e.g., against mycoses). 

Fungal EVs might also become an asset as biosensors. It is believed that in biofilm formation, the EVs can manage both intra- and inter-species communication; they are crucial in maintaining biofilm communities. They could act as radars of opportunist biofilm formation and response to antifungal treatments and in beer or wine fermentation. They might also be used for sensing contamination or monitoring industrial processes such as soil and water remediation. 

Both the basic research into the EVs and their clinical applications would benefit from the development of reference materials (RMs) for EV study. Synthetics monodisperse or polydisperse nanoparticles are used as RMs for EV analysis employing the NTA, DLS or flow cytometry. However, RMs with biochemical and physicochemical resemblance to natural EVs would allow perfect calibration in nano-technique procedures; fungal EVs would be an excellent source of such components.

Nevertheless, we should not forget that basic research in this field is still needed to overcome the current limitations. Moreover, improving our understanding of the EV biogenesis and function will help to identify the EV-dependent mechanisms and use this knowledge in future applications. For example, analysing the role of fungal EVs in the division of labour in *C. gattii* might elucidate the infection mechanisms and help us tackle similar pathologies.

## Figures and Tables

**Table 1:** Protein and nucleic acid composition of EVs in fungi.

Specie	Nucleic Acids	Proteins	References
*Cryptococcus neoformans*	RNA [30,31,32] ncRNA: rRNA, tRNA, smallRNA (miRNA, snRNA, snoRNA) [31,33,34] mRNA [33,35] - Pathogenesis/immune response [33] - Cell-wall remodelling [33] - Metabolism [33] - Translation [33] - Signalling and cell cycling [33] - Redox response [33] - Stress response and nutrition [33] - Transport [33] - Signalling and cell cycling [33] - Traffic and cell organization [33]	**Categories** - Pathogenesis/immune response [14,16,18,19,20,22,29,36,37,38,39,40,41,42,43] - Cell-wall remodelling [11,13,14,16,19,22,29,36,38,39,40,41,42,43,44] - Carbohydrate and lipid metabolism [11,13,14,16,19,22,23,29,36,38,39,40,41,42,43,44,45] - Amino Acid and protein Metabolism [11,13,14,16,19,22,23,29,36,38,39,41,43,44] - Protein Folding [11,13,14,16,19,22,29,36,38,39,41,43,44,46] - Translation [11,13,14,16,22,23,29,36,39,40,41,43,44,45] - Signalling and cell cycling [11,13,14,16,29,36,39,40,41,42,43,44] - Redox response [11,13,14,16,22,23,29,36,40,41,43,44] - Stress response and nutrition [11,13,14,16,19,22,23,29,36,38,39,40,41,42,43,44,46] - Transport [11,13,14,16,22,23,29,36,38,39,40,41,42,43,46] - Traffic and cell organization [11,13,14,16,19,22,23,29,36,38,39,40,41,42,43,44] **Enzymatic activity** - *Laccase, Urease, Phosphatase, Catalase* [13,43] **Infective particles** - *Prions (S*up35p) [37,47]	[10,13,30,33,35]
*Histoplasma capsulatum*			[16,43]
*Paracoccidioides brasiliensis*			[14,17,27,33]
*Malassezia sympodialis*			[18,34,42]
*Candida albicans*			[19,29,33,38,39,40,41,46]
*Sporothrix brasiliensis*			[22]
*Cryptococcus gattii*			[32]
*Exophiala dermatitidis*			[20]
*Saccharomyces cerevisiae*			[23,37,45] * [11,33,37,44,47]
*Pichia fermetans*			[31]
*Fusarium oxysporum*			[36]

* periplasmic vesicles (PVs).

**Table 2:** Kingdom specific component of fungal EVs.

Specie	Carbohydrates	Lipids	Others	References
*Cryptococcus neoformans*	**Polysaccharide** - Glucuronoxylomannan (GXM) [10]	**Categories** - Sterols [43] *Ergosterol* [10,17,19,27] *Obtusifloliol-like* [10] *Brassicasterol* [17,27] *Lanosterol* [17,19,27] - Neutral glycosphingolipid (GSL) *GlcCer* [10,19,27] - Phospholipids [16,27,29] *Phosphatidylethanolamine (PE)* *Phosphatidylserine (PS)* *Phosphatidylcholine (PC)* *Phosphatidic acid (PA)* *Phosphatidylinositol (PI)* *Phosphatidylglycerol (PG)* - Fatty acids [27] *Linoleic acid (C18:2)* *Oleic acid (C18:1)* *Stearic acid (C18:0)* *Palmitic acid (C16:0).* *Pentadecanoic acid (C15:0)*		[10]
*Histoplasma capsulatum*				[16,43]
*Paracoccidioides brasiliensis*	**Polysaccharide** - alpha-linked galactopyranosyl (α-Gal) [17]			[17,27]
*Candida albicans*	**Polysaccharide** *(biofilm)* - *Mannan* [39] - *Glucan* [39]			[19,29,39]
*Cryptococcus gattii*	**Polysaccharide** - Glucuronoxylomannan (GXM) [21,32]			[21,32]
*Malassezia sympodialis*			Allergens	[18,42]
*Sporothrix brasiliensis*			Detected by antiserum	[22]
*Exophiala dermatitidis*			Melanin	[20]
*Fusarium oxysporum*			Detected by antiserum	[36]

**Table 3:** Morphology and profile of fungal EVs.

Specie	Strains	EVs Physic Characteristic	Growth Conditions	References
*Cryptococcus neoformans*	ATCC 24067 (serotype D) [10] HEC3393 (serotype A) [10,33] H99 (serotype A) [10,13,30,33,35] B3501 (serotype D) [30,33] Cap67 (B3501 acapsular) [10,13,30,33] 2E-TUC (serotype D) [13] 2E-TU (2E-TUC *LAC1^-^*) [13] 24067 (serotype D) [30]	- Rounded and bilayered (cryoTEM, resin sections) [10,13] - Ranged (60–300 nm) [10] - Ranged (50–250 nm) and peaks at 300, 410, 500 and 630 nm [35]	1–2 L, minimal medium, 2–3 days (stationary phase), 30 °C, shaking.	[10,13,30,33,35]
*Histoplasma capsulatum*	G217B (ATCC 26032) [16,43]	- Rounded and bilayered (cryoTEM, resin sections) [16] - Ranged (10–350 nm) [16] Small vesicles (40–60 nm) [16,43] Large vesicles (170–250 nm) [16,43] - Abundance: 60% (7–50 nm) > 30% (51–100 nm) > 10% (101–350 nm) [16]	0.5 L, Ham’s F-12/Glc medium, 2–7 days, 37 °C, shaking [16]. **or** 50 mL final, Ham’s F-12/Glc medium, refresh with 10 mL each 2 days for log phase maintenance (3 times, 7 days total), 37 °C, shaking [43].	[16,43]
*Paracoccidioides brasiliensis*	Pb18 (group S1) [14,17,27,33] Pb3 (group PS2) [17,27]	- Rounded and bilayered (cryoTEM, resin sections) [17] - Ranged (20–200 nm) [17]	0.5 L, Ham’s F-12/Glc medium, 4 – 5 + 2 days, 36 °C, shaking.	[14,17,27,33]
*Malassezia sympodialis*	ATCC 42132 [18,34,42]	- Rounded and cup-shaped (negative staining) [18,34,48], (cryo-tomography) [42] - Ranged (50–600 nm) [18,34,48]	2 × 10^6^–60 × 10^6^ cells/mL, 0.3 L, RPMI-1640 or Dixon-MES medium, 2–3 days, 37 °C or 32 °C, shaking.	[18,34,42,48]
*Candida albicans*	11 [19,33] ATCC 90028 [19,40,41] ATCC SC5314 [19,29,38] SN152 [39,46] Clinical isolated [41] DAY286 [40] ATCC 10231 [40]	- Rounded and bilayered (cryoTEM, resin sections) [19,29,38,46], SEM [29,39], negative staining [40,41] - Ranged (30–500 nm) [40,41] Medium vesicles (50–200 nm) [39] Large vesicles > 200 nm (pe 350–450 * nm and 450–850 **, 200–1000 nm) [39]) - *Biofilm*: [4,7,39,40] Medium vesicles (30–400 nm) Large vesicles (500–1200 nm)	Sabouraud medium, 48 h [19,33], or SD medium, 16 h, OD_600_ 4 [38], 30 °C [19,38], or 0.1 L, YPD, 72 h, 37 °C [29], or OD_600_ 0.1, Sabouraud medium, 22 h, 36 °C–37 °C [29], shaking in all cases. **or** RPMI 1640-MOPS (*planktonic* [39] and *biofilm*), 48–54 h, 37 °C [39,40]. **or** OD_600_ 0.2, 0.1–0.3 L (1 L *biofilm*), YPD 0.1, 0.3 or 2% Glc, 20–48 h, 30 °C, shaking [40,46].	[19,29,33,38,39,40,41,46]
*Sporothrix brasiliensis*	5110 (ATCC MYA-4823) [22]	- Rounded and bilayered (cryoTEM, resin sections) - Ranged (50–300 nm): Medium vesicles (50–150 nm) Large vesicles (150–300 nm)	BHI medium, 6 days, 37 °C, shaking.	[22]
*Cryptococcus gattii*	R265 [32]	- Rounded (negative staining) [1] and bilayered (cryoTEM, resin sections) - *In YPD or Sabouraud’s solid medium:* Medium vesicles (100–300 nm) - *In Sabouraud’s solid medium:* Big vesicles (300–600 nm)	0.5 mL YPD [21] or confluent YPD or Sabouraud’s medium (agar) [32], 24–72 h, 25 °C, shaking when liquid.	[32]
*Exophiala dermatitidis*	EXF-10123 [20]	- Rounded (negative staining) - Average radius of 75–90 nm	0.4 L, OD_600_ 0.2, YNB medium, 15 h, 37 °C, shaking	[20]
*Saccharomyces cerevisiae*	BY4742 [23] BY4741 [44,45] RSY225 [11] BY4741 [11] RSY113 [11] SEY6210 [11,33] 74-D694 ± strong ([PSI+]S) or weak ([PSI+]W) Sup35p prion variants, or ± GFP-tagged Sup35p [37,47]	**Periplasmic vesicles** - Rounded and cup-shaped (TEM-negative staining) [23,37] - *In glucose starved cells:* 95% small vesicles (30–50 nm) 5% large vesicles (100–300 nm) - *30 min after glucose:* 4% small vesicles (30–50 nm) 4% large vesicles (100–300 nm) (*with respect the total number of vesicles in starved cells) - *In 74-D694 ± strong ([PSI+]S) or weak ([PSI+]W) Sup35p prion variants, or ± GFP-tagged Sup35p* [37]: 76% small vesicles (<100 nm) 12% large vesicles (200–600 nm) **EVs** - Rounded and bilayered (cryoTEM, resin sections) - Ranged vesicles (50–250 nm): Small vesicles (50–75 nm) [11,33] Median vesicles (100–200 nm) [11] Large vesicles (180–250 nm) [11,33] - *In 74-D694 ± strong ([PSI+]S) or weak ([PSI+]W) Sup35p prion variants, or ± GFP-tagged Sup35p* [37,47] 85% small vesicles (<100 nm) 4% large vesicles (200–600 nm)	40 mL YPKG 0.5% Glc 1–3 d + YPD 2% Glc, 30 min—15 h, 37 °C, shaking [23] *. or 1–2 L, OD_600_ 0.1, YPDA 0.5% or 2% Glc, 3 d, 30 °C, shaking [37] **. **or** Sabouraud dextrose broth, 18–72 h, 25–37 °C, shaking [11] **or** 2 L, OD_600_ ~ 0.1, YPDA, 24 h, 30°C, shaking (final OD600 nm ~ 5–6) [47]. **or** OD_600_ 0.2, YPD, 18 h, 30 °C, shaking[44].	[23,37,45] * [11,33,37,44,47]
*Pichia fermetans*	Lodder [31]	- Rounded (negative staining) - Ranged vesicles (5–60 nm) - *In YCU* --> Diameter ranged from 8–57 nm with an average of 31.2 nm. - *In YCM* --> Diameter ranged from 5–46 nm with an average of 23.7 nm.	5–8 mL, YCU or YCM medium, 48 h, 28 °C, static.	[31]
*Fusarium oxysporum*	VCG01111 [36]	- Rounded (negative staining) - Medium vesicles (100–250 nm)	Liquid cultures were grown in half-strength potato dextrose broth (1/2 PDB), 72 h, 25 °C, shaking.	[36]

Shape and distribution of EV population according to specie, strain and growth conditions. * periplasmic vesicles (PVs), ** both periplasmic (PVs) and extracellular vesicles (EVs).

**Table 4:** Genes involved in biogenesis of fungal EVs.

Gen	Mammalian Ortholog	Function	Phenotype in the Mutant	References
*FKS1*	No cell-wall in mammals.	Catalytic subunit of 1,3-beta-D-glucan synthase. Cell wall remodeling.	- Higher protein content in EVs.	[44]
*CHS1*	No cell-wall in mammals.	Chitin synthase I; Cell wall remodeling and mitotic division septum formation.	- Higher protein content in EVs. - These EVs fail to rescue cells from toxic effects of antifungal agents.	[44]
*CHS3*	No cell-wall in mammals.	Chitin synthase III. Cell wall remodeling.		[44]
*BOS1*	*AtMEMB11/* *AtMEMB12*	v-Snare. Localized to the ER membrane, necessary for vesicular transport from ER to GA.	- Changes in EV protein abundance (not severe).	[11]
*SEC1*	*STXBP1*	Localized to sites of secretion. Involved in docking and fusion of exocytic stimulating membrane fusion.	- Changes in EV protein abundance (severe). - Decreased sterols in EVs accompanied with intracellular accumulation.	[11]
*SEC4*	*RAB8B*	Regulation of polarized delivery of vesicles to the exocyst.	- EVs release and kinetics delayed. - Ranged 100–200 nm EVs pass to two segregated populations of 80–120 and 400–550 nm. - Changes in the protein abundance (severe). - Decreased sterols in EVs accompanied with intracellular accumulation.	[11]
*SEC6*	*EXOC3*	Exocyst complex subunit. Mediation of polarized targeting and tethering of vesicles from GA to active sites of exocytosis at the plasma membrane.	- Ranged 100–200 nm EVs pass to accumulation of 100 nm vesicles in the bud necks of the mutants and large vesicles in the cytoplasm. - Reduction in laccase and urease activity as well as soluble extracellular polysaccharide. Phospholipase activity and GXM molecular architecture unaffected.	[54]
*VPS27 (HSE1)*	*STAM2*	ESCRT-0 complex. Sorting of ubiquitinated membrane proteins into intralumenal vesicles prior to vacuolar degradation and recycling.	- Ranged 100–200 nm EVs pass to larger (>200 nm). - Reduction in laccase and urease activity, melanin production, capsule and virulence. - Accumulation of MVBs and vacuolar fragmentation.	[44,49]
*VPS23* (*STP22*)	*TSG101*	ESCRT-I complex. Sorting of ubiquitin- proteins into the endosome.	- Ranged 100–200 nm EVs pass to deplete those in 30–150 nm and to increase those in 150–500 nm). - Changes in EV protein abundance (not severe): less protein content although enrichment in AAA-domain proteins and in Chs1, Chs3 and Fks1.	[11,44]
*VPS36*	EAP45	ESCRT-II complex. Interaction with ESCRT-I to sort ubiquitin- proteins into the endosome.		[44]
VPS2 (*DID4*)	*CHMP2A*	ESCRT-III complex. Endocytosis, sorting of integral membrane proteins into lumenal vesicles of MVBs and vacuolar enzymes.	- No significant differences in EVs size. - Changes in EV protein abundance (not severe): less protein content although enrichment in AAA-domain proteins and Fks1.	[44]
*VPS32* (*SNF7*)	*CHMP4A/B*	ESCRT-III complex. Sorting from cytoplasm to endosomes and of transmembrane proteins into MVBs.	- No morphological alterations in their EVs. - Changes in the EV protein abundance (severe).	[44]
*BRO1*	Alix/AIP1	Cytoplasmic class E VPS factor. Coordination of deubiquitination in MVBs by recruiting Doa4p to endosomes.	- No significant difference in EVs size. - Similar EV protein content.	[44]
*GRH1* *(GRASP)*	*GRASP65*	GA trafficking and autophagosome formation.	- EV population of 50–250 nm and peaks in 300, 410, 500 and 630 nm pass to range <250 nm with a minor peak at 225 nm and major peaks at 100 and 140 nm. - Required for polysaccharide secretion and virulence factors, proteins and RNA. - Decreased sterols in EVs accompanied with intracellular accumulation.	[35,55]
*END3*	*EPS15*	Endocytosis, actin cytoskeletal organization and cell wall morphogenesis. Vacuolar degradation of plasma membrane proteins.	- Lack of glucose availability sensing. - Lack of EVs/PVs production in response to glucose starvation.	[23] *
*DRS2*(*APT1*)	*ATP8B1*	Putative flippase. Fusion events at GA and plasma membrane.	- EV size pass from 10–150 nm and 400–1000 nm to 10–150 nm and 400–600 nm. - Decreased GXM secretion. - Central localization of the Golgi in the cell.	[56]
*AIM25*	*PLSCR2; PLSCR4; PLSCR1*	Putative scramblase. Regulator of secretion and target for several antifungals.	- EVs within a range of 100–300 nm pass to larger dimensions. - Disorganized membranes in the cell. - Less secretion of EVs with altered RNA profile. - More efficient extraction of EV-GXM, resulting in facilitated capsule enlargement.	[32]
*CHO1*	*PTDSS1/PTDSS2*	Phosphatidylserine synthase. Localized to the mitochondrial outer membrane. Phospholipid biosynthesis	- Most of EVs within the normal range from 50–100 nm. - In *psd1/psd2* doble mutant a new population of >100 nm. - Less secretion of EVs with altered protein profile. - Less protease and phospholipase activity. - Affected mitochondrial function, cell wall thickness and virulence in mice.	[29]
*PSD1*	*PISD*	Phosphatidylserine decarboxylase. Localized to the mitochondrial inner membrane. Phospholipid metabolism and inter-organelle trafficking of phosphatidylserine.		[29]
*PSD2*	*PISD*	Phosphatidylserine decarboxylase. Localized to GA, endosomes and vacuole. Regulation of phospholipids in compartments that will eventually give rise to the vacuole.		[29]
*CLN1*	*CCNA2; CCNB1; CCNC; CCND1; CCNE1*	Cyclin-dependent protein kinase (CDK) regulatory subunit. Regulation of cell cycle.	- Increased capacity to enlarge the capsule. Significant increase in the production of EVs and higher sterol content. Upregulation of the glyoxylate acid cycle.	[57]

* periplasmic vesicles (PVs).

## Abbreviations

EVs	Extracellular vesicles
MVs	Microvesicles
PVs	Periplasmic vesicles
ILVs	Intraluminar vesicles
MVBs	Multivesicular bodies
EE	early endosomes
LE	late endosomes
VC	vacuole
CW	cell wall
CAP	capsule
PM	plasmamembrane
PP	periplasm
ER	endoplasmic reticulum
GA	Golgi apparatus
GRASP	Golgi Reassembly Stacking Protein
IVCs	Intracellular vesicle clusters
Nu	nucleus
GPI-AP	Glycosylphosphatidylinositol (GPI)-anchored proteins
AAA	ATPases Associated with diverse cellular Activities
GXM	Glucuronoxylomannan
sRNA	small RNA
tRNA	transfer RNA
rRNA	ribosomal RNA
miRNA	micro RNA
snRNA	small nuclear RNA
snoRNA	small nucleolar RNA
GSL	neutral glycosphingolipid
GlcCer	glucosylceramide
PE	Phosphatidylethanolamine
PS	Phosphatidylserine
PC	Phosphatidylcholine
PA	Phosphatidic acid
PI	Phosphatidylinositol
PG	Phosphatidylglycerol
